# A case of canine cutaneous pythiosis in Thailand

**DOI:** 10.1099/acmi.0.000109

**Published:** 2020-02-14

**Authors:** Ariya Chindamporn, Patcharee Kammarnjessadakul, Sawang Kesdangsakonwut, Wijit Banlunara

**Affiliations:** ^1^​ Department of Microbiology, Faculty of Medicine, Chulalongkorn University, Bangkok 10330, Thailand; ^2^​ Department of Microbiology, Faculty of Medical Technology, Huachiew Chalermprakiet University, Samut Prakan, 10540, Thailand; ^3^​ Department of Pathology, Faculty of Veterinary Science, Chulalongkorn University, Bangkok 10330, Thailand

**Keywords:** dog, skin, phylogenetic analysis, *Pythium insidiosum*, Thailand

## Abstract

**Introduction:**

*Pythium insidiosum* causes pythiosis in humans and animals in tropical and subtropical climates. The clinical manifestations in humans are mostly systemic, vascular or ocular forms, in contrast to animals, which are cutaneous, subcutaneous and gastrointestinal forms. The highest incidence of human cases is reported in Thailand, however, no canine pythiosis has been documented yet.

**Case presentation:**

A female, mixed-breed, stray dog showed severe extensive ulcerative haemorrhagic dermatitis at the perineum involving the anus and tail. On cytology, there were sparse branching septate fungal hyphae. The tissue samples were subjected to polymerase chain reaction and gene sequencing for fungal identification.

**Conclusion:**

The results of the internal transcribed spacer 1 and 2 (ITS1 and ITS2) gene had 99 % homology to *Pythium insidiosum* (accession no. FJ17396) and the *COX2* gene (accession no. GQ451572). The phylogenetic tree of both genes was classified in clade A_TH._ This is the first fully documented diagnosis of canine cutaneous pythiosis in Thailand.

## Introduction

*Pythium insidiosum*, an oomycetes organism, is the only species in the genus *Pythium* causing destructive malady in humans and animals in tropical and subtropical areas. The clinical manifestations of humans are systemic, vascular and ocular forms. In contrast, the clinical presentations of animal pythiosis are cutaneous, subcutaneous and gastrointestinal forms. Based on accumulated information to date, there have been reports in some livestocks, companion and wild animals. Those infected animal species were bovine, canine, feline, equine and avian (Californian nestling white-faced ibis; *Plegadis chihi*) [1–7]. Also, spectacle bears from zoos were infected [8]. These cases were documented from all continents, mainly, in tropical and subtropical regions such as America: Texas, Costa Rica, Brazil, Argentina; Asia: India, Indonesia, Japan; Australia and the last continent in 2005, was in Africa [9]. Canine cutaneous and subcutaneous forms had been widely reported in the USA and Brazil [2–6, 9–11]. However, an exceptional pulmonary case in a Staffordshire terrier was documented [12]. Even though the first human case in Thailand was reported in 1985 and the highest incidence of human cases is in Thailand, no canine case has been documented yet [13]. Most of these human cases were diagnosed by direct examination or histopathology, showing non-septate hyphae, and the diagnosis was confirmed by rapid growth on the medium and/or molecular techniques. All the *P. insidiosum* isolates documented in Thailand demonstrated that their phylogenetic trees were classified in clade B_TH_ (clade II) and clade C_TH _(clade III) based on the universal primers, named internal transcribed spacer regions (ITS) 2 - ITS 4, and the primers in mitochondrial inner membrane, named the cytochrome oxidase 2 (*COX 2*) gene, which generates the sister clade, providing a better classification over the ITS primers [14]. This is the first canine cutaneous pythiosis pathology report with supporting molecular findings.

## Case report

**Fig. 1.**
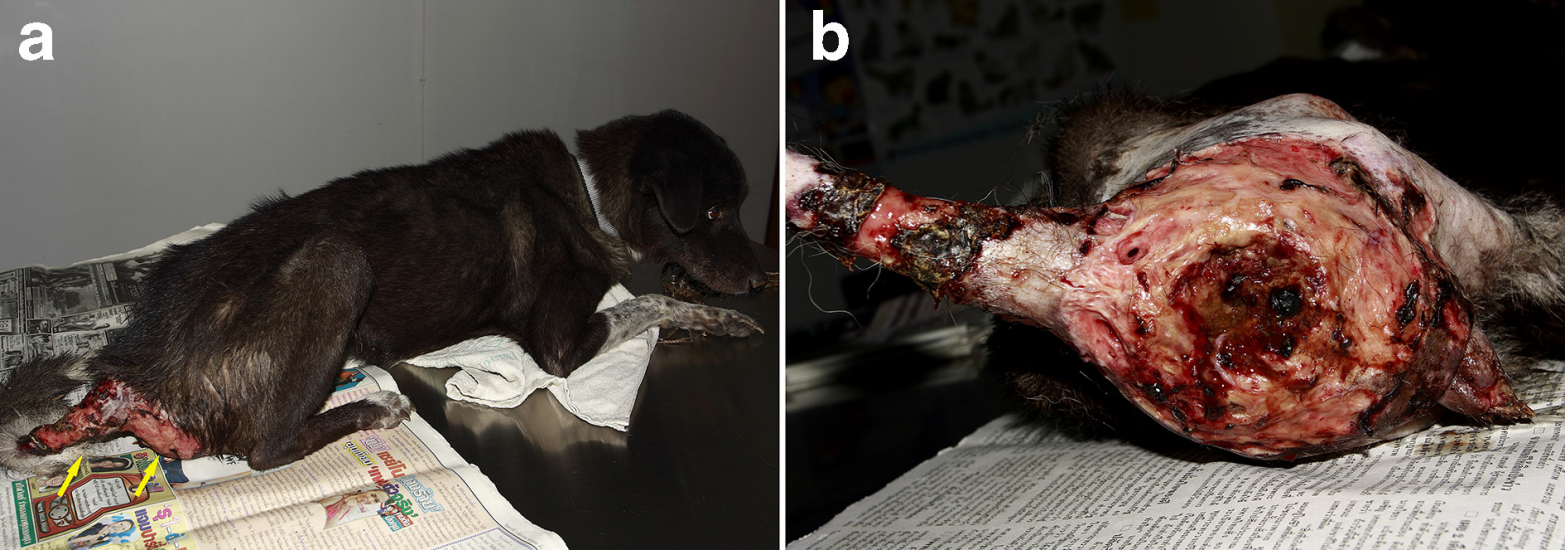
(a) Skin lesions present at the perineum and tail of a dog (arrows). (b) Gross diagnosis is severe extensive ulcerative haemorrhagic dermatitis (left recumbency, posterior view of lesions).

**Fig. 2.**
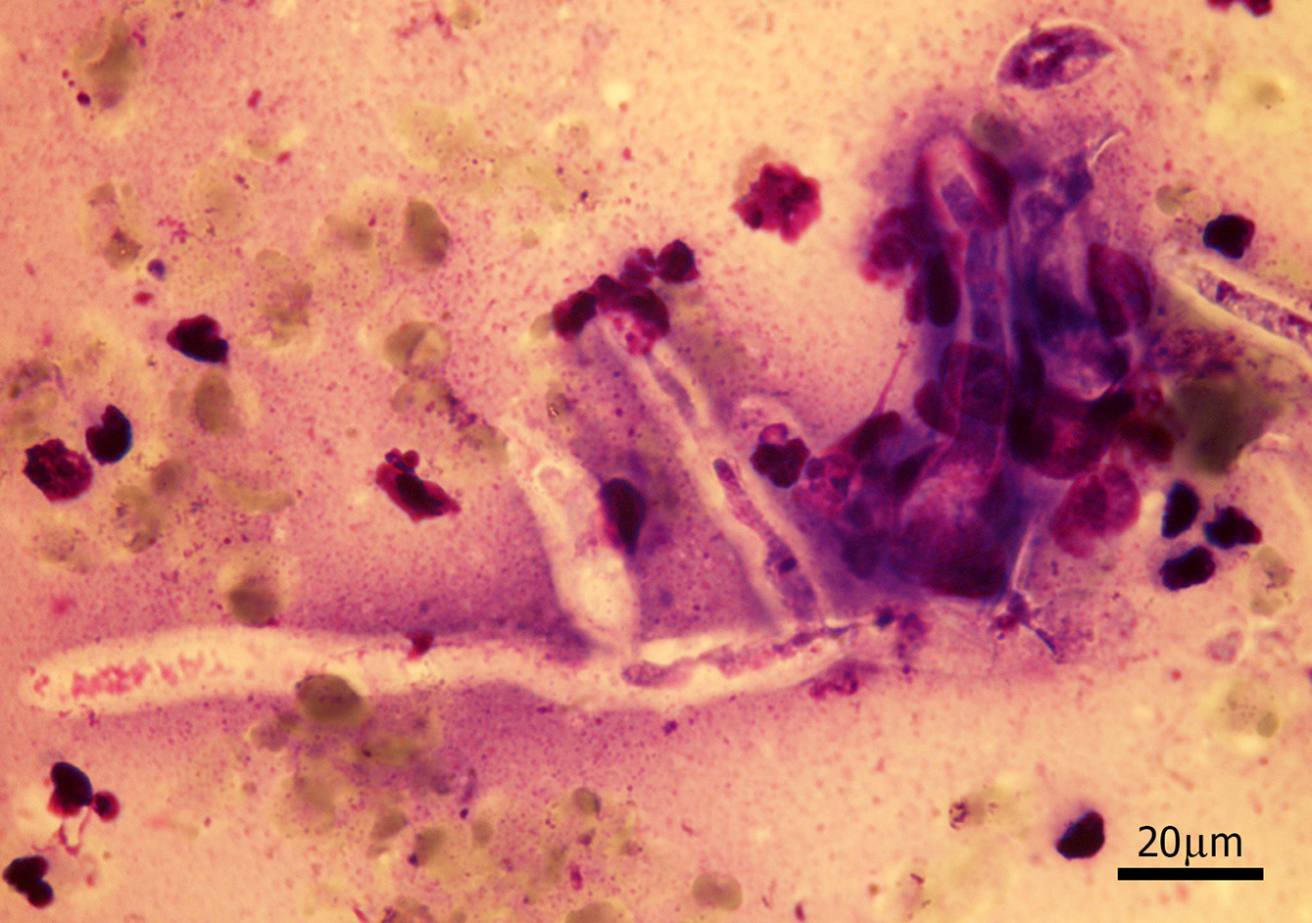
Cytologic finding of the lesion shows contorted branching septate fungal hypha. (Giemsa stain).

**Fig. 3.**
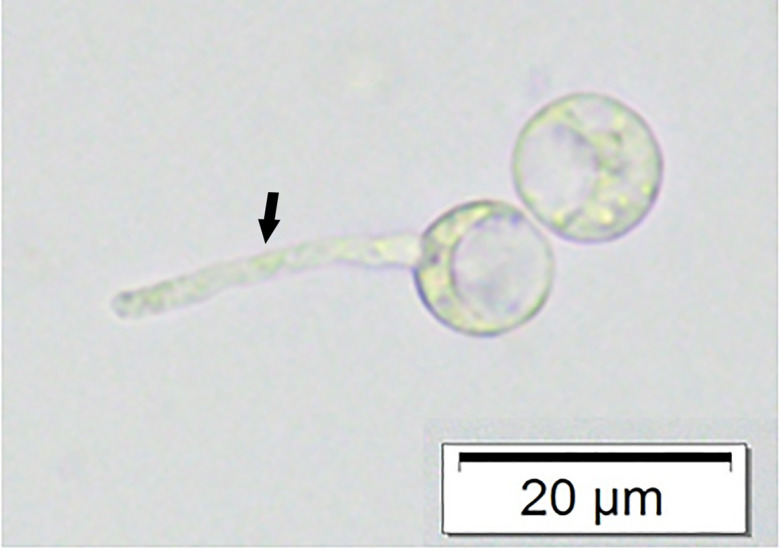
Zoospores of *P. insidiosum* and zoospore germination shows short hypha (arrow) (bar=20 µm).

**Fig. 4.**
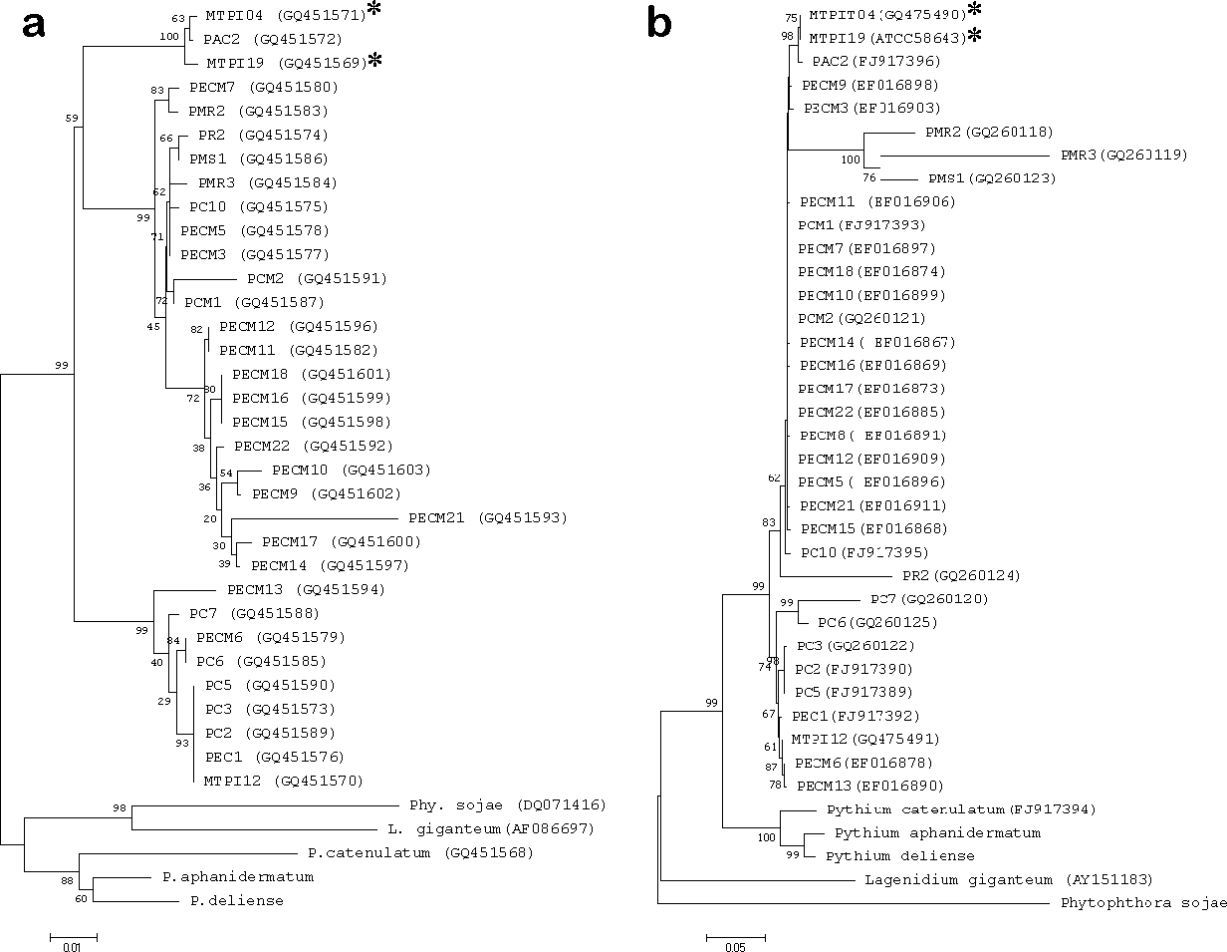
The phylogenetic tree was generated using neighbour-joining (NJ) analysis. Based on 10 000 replicates of bootstrap. (a) COX2 and (b) ITS region (*).

A female, mixed-breed, stray dog, approximately 2–5 years old, was brought to a private small animal hospital in the western vicinity of Bangkok with serious skin lesions, presenting at the perineum, involving the anus and tail. The gross skin lesions showed severe extensive ulcerative haemorrhagic dermatitis (Fig. 1a–b). The cytology samples were prepared from the lesions during the first sampling under the permission of the owner and following the guidelines for the use of animal tissue for the scientific purpose of Chulalongkorn University animal care and use committee. The method used was direct examination using potassium hydroxide (KOH). The rest of the samples were further submitted for fungal culture, and fungal identification using polymerase chain reaction and phylogenetic analysis according to the previously mentioned method [13]. Unfortunately, the animal died after the first visit and necropsy was not allowed. The cytology samples were stained with Giemsa stain. There were eosinophilic contorted septate hyphae, measuring 3–5 μm in diameter (Fig. 2). Similar fungal hyphae were also observed in the submerged creamy rapid growth, 24 h, colony on Sabouraud dextrose agar. In this step, zoospore production was induced and two-flagellate zoospores were demonstrated (Fig. 3). However, the production of zoospores is not the definite identification for *P. insidiosum,* so both the isolate and tissue samples were submitted to amplify the regions of ITS1 and ITS 2 by using polymerase chain reaction. The DNA-sequences of amplicons from both samples showed identical 891 bp. After alignment, these sequences had 99 % homology to *P. insidiosum* (accession no. GQ475490, MTPI04) and its accession number is FJ17396, strain PAC2. The sequences of the isolate from the 550 bp. *COX2* amplicon was identified as *P. insidiosum* (accession no. GQ451572, strain PAC2). Each amplicon was analysed and used to construct the phylogenetic tree (Fig. 4a, b). Both sequences belonged to clade A_TH_.

## Discussion

It is interesting that both sequences belonged to clade A_TH_. This study is the first documented canine cutaneous pythiosis case, which was infected by clade A_TH_. From our knowledge up to the present, the members in clade A_TH_ are isolates from the USA, but not from Asia [15]. It is in contrast to the clades from the environment and all clinical isolates in Thailand, which belong to clade B_TH _and C_TH_ [13, 14]. This report will draw the attention that not only human cases, but also animal cases, which pythiosis should be included as endemic in Thailand. This is a new knowledge of clade A_TH_ *P. insidiosum* occurrence in Thailand. However, the limitation of this case was the exact history.
